# Global report on preterm birth and stillbirth (2 of 7): discovery science

**DOI:** 10.1186/1471-2393-10-S1-S2

**Published:** 2010-02-23

**Authors:** Michael G Gravett, Craig E Rubens, Toni M Nunes, the GAPPS Review Group

**Affiliations:** 1 Department of Obstetrics and Gynecology, University of Washington School of Medicine, Seattle, WA, USA; 2 Global Alliance to Prevent Prematurity and Stillbirth, an initiative of Seattle Children's, Seattle, WA, USA; 3 Department of Pediatrics at University of Washington School of Medicine, Seattle, Washington, USA

## Abstract

**Background:**

Normal and abnormal processes of pregnancy and childbirth are poorly understood. This second article in a global report explains what is known about the etiologies of preterm births and stillbirths and identifies critical gaps in knowledge. Two important concepts emerge: the continuum of pregnancy, beginning at implantation and ending with uterine involution following birth; and the multifactorial etiologies of preterm birth and stillbirth. Improved tools and data will enable discovery scientists to identify causal pathways and cost-effective interventions.

**Pregnancy and parturition continuum:**

The biological process of pregnancy and childbirth begins with implantation and, after birth, ends with the return of the uterus to its previous state. The majority of pregnancy is characterized by rapid uterine and fetal growth without contractions. Yet most research has addressed only uterine stimulation (labor) that accounts for <0.5% of pregnancy.

**Etiologies:**

The etiologies of preterm birth and stillbirth differ by gestational age, genetics, and environmental factors. Approximately 30% of all preterm births are indicated for either maternal or fetal complications, such as maternal illness or fetal growth restriction. Commonly recognized pathways leading to preterm birth occur most often during the gestational ages indicated: (1) inflammation caused by infection (22-32 weeks); (2) decidual hemorrhage caused by uteroplacental thrombosis (early or late preterm birth); (3) stress (32-36 weeks); and (4) uterine overdistention, often caused by multiple fetuses (32-36 weeks). Other contributors include cervical insufficiency, smoking, and systemic infections. Many stillbirths have similar causes and mechanisms. About two-thirds of late fetal deaths occur during the antepartum period; the other third occur during childbirth. Intrapartum asphyxia is a leading cause of stillbirths in low- and middle-income countries.

**Recommendations:**

Utilizing new systems biology tools, opportunities now exist for researchers to investigate various pathways important to normal and abnormal pregnancies. Improved access to quality data and biological specimens are critical to advancing discovery science. Phenotypes, standardized definitions, and uniform criteria for assessing preterm birth and stillbirth outcomes are other immediate research needs.

**Conclusion:**

Preterm birth and stillbirth have multifactorial etiologies. More resources must be directed toward accelerating our understanding of these complex processes, and identifying upstream and cost-effective solutions that will improve these pregnancy outcomes.

## Background

As succinctly stated by Romero et al., "Few biological processes as central to the survival of a species as parturition are so incompletely understood" [1]. This is especially relevant for understanding mechanisms associated with preterm birth and stillbirth. Unfortunately, this lack of knowledge about the process of giving birth has led to largely empirical and ineffective interventions.

**Figure 1:**
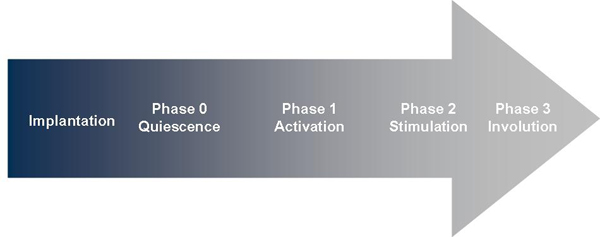
Phases of parturition as a continuum of pregnancy

Two compelling principles emerge from the current understanding of pregnancy and parturition. First, labor represents a natural continuum of processes that begin at implantation and culminate with the return of the uterus to its non-pregnant state [2,3]. Parturition proceeds through well-defined phases (Figure 1): 

•* Implantation* of the blastocyst within the endometrium and characterized by embryonic trophoblast invasion of maternal spiral arteries, allowing establishment of placentation

•* Uterine quiescence,* during which embryogenesis and fetal growth occur and the uterus increases dramatically in size through hypertrophy

•* Activation* of the myometrium, during which cellular and biochemical events occur that promote uterine contractility

•* Stimulation,* or the onset of regular uterine contractions characteristic of labor and birth

•* Involution,* during which the uterus reduces in size and returns to its non-pregnant state—abnormalities in uterine involution are associated with maternal postpartum hemorrhage, a leading cause of maternal mortality globally

The overwhelming majority of pregnancy is spent in uterine quiescence or in activation. Less than 0.5% of pregnancy is spent in active labor, yet most interventions and research have focused on treatment of preterm labor or other intrapartum events. As noted below, it is likely that research directed at understanding the mechanisms maintaining uterine quiescence and the mechanisms of activation that allow the uterus to contract will have significant impact upon the development of rational and efficacious prevention strategies.

The second compelling principle is that preterm birth and stillbirth are complex outcomes with multifactorial etiologies. Preterm birth and stillbirth represent final common outcomes from a wide variety of causes, each with distinct biologic pathways [4,5]. Unfortunately, all preterm births or stillbirths have usually been defined as a single endpoint, regardless of etiology, for epidemiological purposes. This has led to uniform and largely unsuccessful treatments or interventions. In fact, the etiologies of preterm births and stillbirths differ according to gestational age, ethnicity, and characteristics unique to each population. For example, intrauterine infection and decidual hemorrhage are important causes of extreme prematurity and of stillbirth [6], while uterine overdistension associated with multiple gestations and maternal or fetal stress are important causes of preterm birth from 32 to 36 weeks of gestation.

Regardless of initiating factors, these pathways ultimately lead to activation of the fetal membranes and maternal decidua, resulting in common mediators such as prostaglandins and metalloproteinases that stimulate contractility and rupture of the fetal membranes. The finding that heterogeneous origins result in common downstream biological pathways and outcomes provides the opportunity to develop rational treatment strategies that target the unique upstream initiating event and the common downstream effectors [4].

Finally, disparities exist in medically-indicated preterm birth secondary to maternal illness or fetal compromise. In high-income countries, the greatest increases in preterm birth rates have occurred among late preterm births from 34 to 37 weeks of gestation, which now account for 60%-70% of all preterm births [7]. Much of the increase in late preterm births has been attributed to the increased prevalence of multiple gestations associated with assisted reproductive technologies and with medically-indicated preterm birth. Medically-indicated preterm birth is the most rapidly increasing cause of preterm birth in high-income countries, responsible for 30% of all preterm births [7]. In the United States, medically indicated preterm birth increased by 55% from 1989 through 2000 [8]. However, in resource-poor low- and middle-income countries where fetal and maternal well-being assessment technology is not available, medically-indicated preterm birth accounts for less than 10% of preterm births. One consequence of this disparity has been a reduction in stillbirths and perinatal mortality, but a paradoxical increase in preterm births among high-income countries.

Events characterizing both spontaneous parturition and preterm parturition, and critical gaps in the understanding of these processes are described in the following sections.

### Spontaneous term parturition

Understanding events surrounding normal parturition at term is necessary for an informed discussion of pathologic mechanisms leading to preterm parturition. Following implantation, the process of normal spontaneous parturition can be divided into the phases described below in Figure 1(modified from [3,9]).

Pregnancy proceeds through a series of orderly phases, beginning with implantation of the early blastocyst and culminating with delivery and return of the uterus to the non-pregnant state. Perturbations in any of these steps may cause adverse pregnancy outcomes.

#### Implantation

Implantation is characterized by embryonic cytotrophoblast invasion of the endometrium and maternal spiral arteries and is necessary to establish normal placentation. This process is facilitated by maternal immunologic processes that involve uterine natural killer monocytes (NK cells) and a shift from a pro-inflammatory to an anti-inflammatory intrauterine milieu. Perturbations in implantation have been associated with habitual abortions, stillbirths, and with preeclampsia, which is an important cause of medically-indicated preterm birth [10].

#### Phase 0: Quiescence

Throughout the majority of pregnancy the uterus remains relaxed and quiescent. Myometrial activity is inhibited by a variety of biologic compounds including progesterone, nitric oxide, and relaxin. Rare uterine contractions during the quiescent phase are of low frequency and amplitude and are poorly coordinated throughout the uterus; these are commonly referred to as Braxton-Hicks contractions in women. The poor coordination of these contractions is due to an absence of gap junctions and contractile-associated proteins that otherwise allow direct cell-to-cell coupling of electrical signaling [11].

#### Phase 1: activation

Myometrial activation occurs in Phase 1 and is characterized by increased expression of myometrial contractile-associated proteins and cellular receptors for oxytocin and prostaglandins, both of which stimulate uterine contractility [12]. The signals for myometrial activation come from uterine stretch (which induces contractile-associated protein and oxytocin gene expression) and from maturation of the fetal hypothalamic-pituitary-adrenal (HPA) axis, as characterized below.

#### Phase 2: stimulation

Phase 2 is a progressive cascade of events leading to a common pathway of parturition involving high amplitude, regular uterine contractions, cervical ripening, and activation of the decidua and fetal membranes. Estrogens, produced by the placenta play a central role in the coordination of these events. Estrogens increase the expression of many contraction-associated proteins, including connexin 43, and of oxytocin and prostaglandin receptors; estrogens also promote prostaglandin production and increases myosin light-chain kinase (MLCK, which stimulates myometrial contractions) [3]. These changes promote myometrial contractility. Additionally, there is a shift in progesterone receptor (PR) isoforms from the normally dominant PR-B to a truncated, inactive PR-A, leading to a state of functional progesterone withdrawal that promotes myometrial contractility. Placental estrogen production is dependent upon precursor fetal adrenal androgens that are aromatized by placental steroid aromatase into estrogens. Thus, the events of Phase 2 are characterized by maturation and activation of the fetal HPA axis. The events leading to fetal HPA activation are incompletely understood but it is thought that placental corticotrophin-releasing hormone (CRH) plays a central role [13]. CRH, a neuropeptide of predominantly hypothalamic origin, is also expressed in the human placenta and membranes and released in exponentially increasing amounts over the course of gestation into maternal and fetal compartments. The trajectory of CRH rise has been associated with the length of gestation [14]. These findings have led some researchers to suggest that placental CRH may act as a "placental clock" and regulate the length of gestation [14]. Placental CRH synthesis is stimulated by adrenal glucocorticoids. Placental CRH, in turn, promotes fetal cortisol and androgen production, and this positive feedback loop is progressively amplified, thereby driving the process forward from fetal HPA activation to estrogen biosynthesis and parturition.

Cervical softening and decidual and fetal membrane activation also occur during Phase 2. Cervical ripening is characterized by a decrease in total collagen content, an increase in collagen solubility, and an increase in collagenolytic activity that results in the remodeling of the extracellular matrix of the cervix [15]. Prostaglandins, estrogens, progesterones, and inflammatory cytokines all promote extracellular matrix metabolism and cervical ripening. Decidual and fetal membrane activation refers to a complex set of anatomical and biochemical events eventually resulting in the rupture of membranes. The precise mechanism of the decidual and fetal membrane activation is not yet known, but extracellular matrix-degrading enzymes such as matrix metalloproteinase 1 (MMP-1), interstitial collagenase, MMP-8 (neutrophil collagenase), MMP-9 (gelatinase-B), neutrophil elastase, and plasmin have been implicated. These enzymes degrade extracellular matrix proteins (e.g., collagens and fibronectins), thereby weakening the membranes and eventually leading to the rupture of membranes.

#### Phase 3: involution

Phase 3 begins with the third stage of labor and involves placental separation and uterine contraction. Placental separation occurs by cleavage along the plane of the decidua basalis. Uterine contraction is essential to prevent bleeding from large venous sinuses that are exposed after delivery of the placenta, and is primarily affected by oxytocin. Postpartum hemorrhage, an abnormality of Phase 3, is a leading cause of maternal mortality worldwide.

### Summary of spontaneous term parturition

Parturition involves a progressive cascade of events initiated by HPA activation and increased placental CRH expression, leading to a functional progesterone withdrawal and estrogen activation, which results in the expression and activation of contraction-associated proteins (CAPs), including oxytocin, and prostaglandin receptors. This biological cascade eventually leads to a common pathway involving cervical ripening, uterine contractility, decidual and fetal membrane activation, and, in the second stage, increases in maternal oxytocin. It has been hypothesized that both preterm and term labor share this common pathway and that pathological stimuli of parturition, as described in the following sections, may act in concert with the normal physiological preparation for labor, especially after 32 weeks of gestation. Prior to 32 weeks of gestation, a greater degree of pathological stimulus may be required to initiate labor.

One fundamental difference between the spontaneous parturition at term and preterm labor is that term labor results from physiological activation of components of the common pathway, while preterm labor arises from pathological processes that activate one or more of the components of the common pathway of parturition. However, further research is necessary to answer fundamental questions, including: 1. What are the mechanisms that maintain uterine quiescence for greater than 95% of the total length of gestation? 2. What is the basis for the disparities in gestational length and risks of preterm birth between ethnic and socioeconomic groups? 3. Do they have a biologic basis, or can they be accounted for by environmental factors?

## Pathways to spontaneous preterm birth

Preterm birth may result from preterm labor with intact fetal membranes, preterm rupture of the fetal membranes, or from iatrogenic preterm delivery for maternal or fetal indications. In high-income countries, approximately 40-45% of preterm births follow preterm labor, 25-40% follow preterm premature rupture of the fetal membranes, and 30-35% are indicated deliveries [7]. In contrast, studies from countries in Latin America have shown that almost 70% are spontaneous preterm births, 16-21% involve rupture of membranes, and 11-15% have medically induced causes [16,17].

Until recently, a tendency has existed among obstetricians and epidemiologists to combine, for statistical purposes, all preterm births occurring between 22 and 37 weeks of gestation. The traditional empirical approach to preterm labor presupposed a single pathologic process for which treatment could be uniform. It is now clear the causes of preterm labor are multifactorial and vary according to gestational age, genetic, and environmental factors. A useful paradigm for pathologic pathways contributing to preterm birth has been provided by Lockwood and Kuczynski [18]. These pathways include systemic and intrauterine infection, uteroplacental thrombosis and intrauterine vascular lesions or decidual hemorrhage, stress, and uterine overdistension (Table 1). While each of these may cause preterm birth at any point in gestation, infection/inflammation predominates as a cause of early preterm birth (24-32 weeks gestation), and stress and uterine overdistension are associated mostly with late preterm birth (32-37 weeks).

**Table 1:** Commonly recognized etiologies and pathways leading to spontaneous preterm birth

Pathway	Examples	Mechanistic Effectors	Gestational Age When Predominant
Infection or Inflammation	Intrauterine Lower genital tract Systemic	Pro-inflammatory cytokine/ prostaglandin cascade Matrix metalloproteinases	Early preterm birth (24-32 weeks)
Decidual Hemorrhage	Thrombophilias, Placental abruption Autoantibody syndromes	Thrombin Matrix metalloproteinases	Early or late preterm birth
Maternal/Fetal HPA Activation	Stress	Maternal/Fetal HPA activation Placental CRH Estrogens Immune modulation	Late preterm birth (32-36 weeks)
Pathologic Uterine Overdistension	Multifetal gestation Polyhydramnios	Expression of gap junctions proteins Prostaglandins Oxytocin receptors	Late preterm birth

Additionally, each pathway may be influenced by gene-environment interactions and by genetic variability in single nucleotide polymorphisms, described below. Commonly occurring pathways of preterm parturition are described below.

### Infection/inflammation

Infections are strongly related to preterm birth [19]. Infectious sources linked to preterm birth include intrauterine infections, lower genital tract infections, and systemic maternal infections.

Intrauterine infection is recognized as one of the most important and potentially preventable causes of early preterm birth. These infections are thought to be responsible for up to 50% of extreme preterm births of less than 28 weeks of gestation, where both neonatal mortality and morbidity are high. The prevalence of microbial invasion of the chorioamnion is 73% in women with a spontaneous preterm birth prior to 30 weeks of gestation, and only 16% among women with indicated preterm delivery without labor [20]. One sentinel study found the frequency of intrauterine infection with recovery of micro-organisms from amniotic fluid to be 45% at 23-26 weeks of gestation, 16% at 27-30 weeks, and 11% at 31-34 weeks [21]. Intrauterine infection as a cause of preterm birth is rare beyond 34 weeks of gestation. It is likely that recent microbiology techniques such polymerase chain reaction identification of fastidious microorganisms will lead to even greater estimates of infection as a cause of preterm birth.

Furthermore, a high proportion of women in preterm labor with evidence of microbial invasion of the amniotic fluid are refractory to standard tocolytic therapy and experience rapid preterm delivery (62% versus 13% of women with sterile amniotic fluid) [22]. This suggests the pathophysiology of infection-associated preterm labor differs from that of idiopathic preterm labor. There is now considerable evidence to suggest the pro-inflammatory cytokine/prostaglandin cascade plays a central role in the pathogenesis of infection-associated preterm birth [23]. These inflammatory mediators are produced by macrophages, decidual cells, and fetal membranes in response to bacteria or bacterial products. A role for selected cytokines in preterm labor is based upon the following observations: elevated amniotic fluid concentrations of cytokines and prostaglandins are found in patients with intra-amniotic infection and preterm labor; *in-vitro,* bacterial products stimulate production of pro-inflammatory cytokines by human decidua; these cyto- kines, in turn, stimulate production of prostaglandins by amnion and decidua; administration of interleukin-1 to pregnant mice or non-human primates induces preterm labor which can be prevented by administration of Il-1 receptor antagonist protein.

There is also evidence that lower genital tract infections, especially bacterial vaginosis, or maternal systemic infections such as periodontitis, malaria, and syphilis contribute to preterm birth (see article 3 on interventions), as briefly reviewed below relevant to pathophysiology.

#### Bacterial vaginosis and periodontitis

Observational studies show increased risks of intra-amniotic infection, preterm delivery delivery, low birth weight, and endomyometritis in the presence of bacterial vaginosis (BV) [24,25]. Generally, the presence of BV increases the risk of preterm birth by 50%. However, most women with BV do not have a preterm birth and randomized clinical trials assessing treatment of BV for the prevention of preterm delivery have been disappointing, although treatment of women prior to 20 weeks may be beneficial [26]. These are reviewed in article 3.

Similarly, observational studies have suggested an association between preterm birth or other adverse pregnancy outcomes and periodontal disease [27]. However, a recent meta-analysis of 44 studies, including five controlled trials, found no association between periodontal disease and preterm birth although low- income countries had higher trends towards association [28]. Heterogeneity between studies was noted, such as inconsistent disease and outcome definitions, inadequate control for confounders and insufficient sample size, making firm conclusions difficult to make [28].

The associations between BV or periodontal disease and preterm birth, and the reasons for discrepancy in treatment trials are important to understand, since both BV and periodontal disease occur commonly in pregnancy. Bacterial vaginosis is seen in up to 30% of pregnant women [24]. In high-income countries it is estimated that about 15% of adults between 21-50 years have severe periodontitis [29]. The proportion may be even higher in low-income-countries. Explanations for these discrepancies may include (1) gestational age at diagnosis/ enrollment; (2) past reproductive history; and (3) choice of treatment. Recent research indicates that both BV and periodontitis are complex microbiological processes, involving a wide variety of both cultivable and fastidious microorganisms not previously identified that may be a part of the normal microflora [30,31]. Further, both BV and periodontal are associated with perturbations in the host inflammatory response, characterized by a pro-inflammatory state. These differences have led some to hypothesize that only a subgroup of women harboring certain microbes associated with BV or periodontal disease, or with genetic differences in inflammatory responsiveness may be at risk for preterm birth. These women may have an abnormal inflammatory response to changes in the vaginal ecosystem (either hypo- or hyper- responsive) predisposing them to preterm delivery . In support of this concept, Macones, et al, recently reported that women with the polymorphism coding for TNFα-308 allele that leads to up-regulation of TNF-α are at increased risk of preterm delivery associated with BV [32]. An understanding of the relationships among the human microbiome, host inflammatory responsiveness and pregnancy outcome represents a critical research need.

#### Malaria

Observational studies have demonstrated an association between malaria and risk of preterm birth with odds ratios ranging between 2 and 3 [33-36], although most randomized trials of malaria prevention or treatment do not report specifically on preterm births. However, the evidence of impact on low birth weight is strong, as reviewed in article 3. The magnitude of effect on preterm delivery appears to be based on a number of factors, including timing of infection [33,37], underlying parity [38], severity of infection [33], increasing placental parasitemia [39], and local transmission rates [35]. Placental malaria is also significantly associated with a higher risk for stillbirth (see article 3 on interventions).

Erythrocytes infected with* P. falciparum* differ in important ways from non-pregnant individuals. Placental sequestration occurs in the intervillous space. Placental infection generates an immune response that is characterized by monocytic infiltrates in the placental intervillous space and a change in cytokine balance from Th-2 to Th-1 (specifically increased levels of TNF-α, INF-y, and IL-1P). Elevated levels of these cytokines, particularly TNF-α, have been associated with low birth weight and preterm delivery [40].

#### Syphilis

Syphilis, caused by* Treponema pallidum,* is a risk factor for both stillbirth and preterm birth (see article 3 on interventions). It is estimated to be responsible for 460,000 abortions or stillbirths per year with higher rates in low-income countries [41]. The study of* T. pallidum* is hindered by the fact that it cannot be cultivated for sustained periods using artificial media. However, the complete genome sequence of* T. pallidum* has been published within the past 10 years and provides opportunities for new insight into its pathogenesis. Little is known about its mechanism of action or determinants of virulence [42].* Treponema* are able to cross the placenta and cause infection in the fetus [43]. Primary and secondary syphilis in pregnancy will lead to fetal infection in virtually all cases, with approximately 30-50% of pregnancies resulting in stillbirth or death shortly after delivery [43]. In contrast, fetal infection is less common with tertiary syphilis. The immune response to* T. pallidum* in pregnancy has not been vigorously studied, but the dampening of the intensity of the innate and cell-mediated immune response to favor the maintenance of the fetus may result in incomplete clearance and the development of chronic infection and adverse pregnancy outcome [43]. However, the nature and role of the maternal-fetal immune response, their interactions, and effect on pregnancy outcome remains poorly understood [44].

### Decidual hemorrhage/thrombosis

Decidual hemorrhage may cause either late or early preterm birth. Vascular lesions of the placenta are commonly associated with preterm birth and preterm premature rupture of membranes (PPROM). Vascular lesions of the placenta have been reported in 34% of women with preterm delivery, 35% of women with PPROM, and in 12% of term uncomplicated deliveries [45]. These lesions may be characterized as failure of physiologic transformation of the spiral arteries, atherosis, and maternal or fetal arterial thrombosis. The proposed mechanism linking vascular lesions to preterm birth is related to utero-placental ischemia. Although the pathophysiology remains unclear, thrombin is thought to play a central role.

Independent of its critical role in coagulation, thrombin is a multifunctional protease that elicits contractile activity of vascular, intestinal, and myometrial smooth muscle. Thrombin stimulates increases in basal tone and phasic contractions in longitudinal myometrial smooth muscle* in vitro,* in a dose-dependent manner [46]. Recently, these* in vitro* observations have been confirmed with an* in vivo* model utilizing thrombin and myometrial contractility could be significantly reduced by the addition of heparin, a known thrombin inhibitor. These* in vitro* and* in vivo* experiments provide a possible mechanistic explanation for increased uterine activity clinically observed in abruption placentae and preterm birth following first or second trimester bleeding.

A relationship between thrombin and PPROM may also exist. Matrix metalloproteinases (MMPs) break down the extracellular matrix of the fetal membranes and the choriodecidua and contribute to PPROM, as discussed below.* In vitro,* thrombin significantly increases MMP-1, MMP-3, and MMP-9 protein expression in decidual cells and fetal membranes collected from uncomplicated term pregnancies [47-49]. Thrombin also elicits a dose- dependent increase in decidual interleukin-8, a chemo- attractant cytokine responsible for neutrophil recruitment [50]. Overt placental abruption, an extreme example of decidual hemorrhage, is also associated with a marked decidual infiltration of neutrophils, a rich source of proteases and matrix metalloproteinases [50]. This may provide a mechanism for premature rupture of the membranes (PROM) in the setting of decidual hemorrhage.

### Maternal or fetal HPA activation: stress

Stress results in preterm activation of the maternal or fetal HPA axis and is increasingly recognized as an important cause of late preterm birth. Stress may be simply defined as any challenge, whether physical or psychological, that threatens or is perceived to threaten homeostasis of the patient. Several studies have found 50% to 100% increases in preterm birth rates associated with maternal stress, usually defined as a composite of life events, anxiety, depression, or perceived stress [51]. Neuroendocrine, immune, and behavioral processes (such as depression) have been linked to stress-related preterm birth. However, the most important processes linking stress and preterm birth are neuroendocrine, resulting in preterm activation of the HPA axis. These processes are mediated by placental CRH (reviewed in [52]).* In vitro* studies of human placental cells have shown CRH is released from cultured human placental cells in a dose-response manner to all major biological effectors of stress, including cortisol, catecholamines, oxytocin, angiotension II, and interleukin-1.* In vivo* studies have also found significant correlations between maternal psychosocial stress and maternal plasma levels of CRH, ACTH, and cortisol. Several studies have related early maternal plasma CRH to the timing of birth. Hobel and colleagues [53] conducted serial assessments of CRH over the course of gestation and found that compared with women delivering at term, women delivering preterm had significantly elevated CRH levels as well as a significantly accelerated rate of CRH increase over the course of their gestation. In addition, they found that maternal psychosocial stress levels at mid-gestation significantly predicted the magnitude of increase in maternal CRH levels between mid-and later-gestation.

These data suggest the relationship between maternal psychological stress and prematurity may be mediated by prematurely increased expression of placental CRH. In term parturition, placental CRH activation is driven largely by the fetal HPA axis in a forward feedback loop upon fetal maturation. In preterm parturition the maternal HPA axis may drive placental CRH expression [52]. Maternal stress, in the absence of other mediators of preterm birth such as infection, results in increased levels of biological effectors of stress including cortisol and epinephrine, which could activate placental CRH gene expression. Placental CRH, in turn, can stimulate fetal secretion of cortisol and DHEA-S (via activation of fetal HPA axis) and placental synthesis of estrogens and prostaglandins, thereby precipitating preterm delivery (reviewed in [54]). Stress may contribute to the increased rates of preterm birth observed among African-Americans in the United States.

Asphyxia may represent a common final outcome for a variety of pathways that include stress, hemorrhage, preeclampsia, and infection. Asphyxia plays an important role in preterm birth, stillbirth, and adverse neonatal developmental sequelae. Chronic asphyxia—associated with insufficiency of the utero-placental circulation— may occur in placental infection such as malaria, or maternal illnesses (e.g., diabetes, preeclampsia, chronic hypertension), and is characterized by activation of the fetal HPA axis and subsequent preterm delivery.

### Uterine overdistension

Uterine overdistension plays a key role in the onset of preterm labor associated with multiple gestations, polyhydramnios, and macrosomia. Multiple gestations, frequently attributable to assisted reproduction technologies (ART) including ovulation induction and *in-vitro* fertilization, are one of the most important causes of late preterm birth in high-income countries (HICs). In the United States, for example, ART accounts for 1% of all live births, but 17% of all multiple births; 53% of neonates conceived as a result of ART in 2003 were multiples [4]. The mechanisms whereby uterine overdistension might lead to preterm labor are incompletely understood, and appropriate animal models are lacking. Uterine stretch induces expression contraction-associated proteins such as oxytocin receptors and connexin-43 [55,56].* In vitro* stretch of myometrial strips also increases prostaglandin H synthase 2 (PGHS-2) and prostaglandin E [57]. Stretching the muscle of the lower uterine segment has been shown to increase interleukin-8 (IL-8) and collagenase production, which in turn facilitates cervical ripening [58,59]. However, experimental animal models for uterine overdistension do not currently exist, and human studies have been entirely observational in nature. This is a critical gap in our understanding, given the increasing prevalence of multiple gestations in HICs.

## Cervical insufficiency

Cervical insufficiency has traditionally been associated with second trimester pregnancy losses, but recent evidence suggests that cervical disorders are associated with a wide variety of adverse pregnancy outcomes, including early preterm birth [60-62]. Cervical insufficiency has traditionally been identified among women with a history of recurrent mid-trimester pregnancy losses in the absence of recognized uterine contractions. There are five recognized or possible causes: (1) congenital disorders; (2)* in-utero* diethylstilbestrol exposure (3) loss of cervical tissue following a surgical procedure such as Loop Electrosurgical Excision Procedure (LEEP) or conization; (4) traumatic damage; and (5) infection.

Traditionally, women with a history of cervical insufficiency were offered cervical cerclage in early pregnancy. However, it has been proposed, and it is most likely, that most cases of cervical insufficiency represent a continuum of premature tissue remodeling and cervical shortening from other pathological processes for which cerclage may not always be appropriate and are better predicted by cervical length determined by transvaginal ultrasonography [60]. Cervical length measured by transvaginal ultrasound is inversely correlated with risk of preterm birth [61]; 50% of women with a cervical length of 15 millimeters or less at 22-24 weeks deliver prior to 32 weeks of gestation [63]. Further, there is a correlation between the length of a previous gestation resulting in preterm birth and cervical length in the next pregnancy, but no correlation with an obstetrical history of cervical insufficiency and cervical length in the next pregnancy [60].

These data suggest that the true cervical insufficiency occurs infrequently, and a short cervix more frequently occurs as a consequence of premature cervical remodeling as a result of a pathological process. Infection and inflammation likely play a significant role in cervical shortening and premature dilation. Fifty percent of patients evaluated by amniocentesis for mid-trimester asymptomatic cervical dilation and 9% of patients with a cervical length of <25 millimeters but without cervical dilation have evidence of intra-amniotic infection[64,65]. These data suggest an important role for ascending intrauterine infection, as outlined above, in the short cervix and cervical insufficiency.

## Preterm premature rupture of the fetal membranes

Regardless of the etiology or mechanistic pathway to spontaneous preterm labor, preterm birth is usually preceded by preterm premature rupture of the fetal membranes. Preterm premature rupture of membranes (PPROM) accounts for 25-40% of preterm births [7,66] and represents a final common pathway to preterm birth. Thus, an understanding of the mechanistic pathways leading to PPROM is important in understanding the biologic basis of prematurity. Preterm pre-labor rupture of membranes has been associated with intrauterine infection, tobacco use, abruption, multiple gestations, previous PPROM, previous cervical surgery or laceration, a short cervix by ultrasound, genetic connective tissue disorders, and vitamin C deficiency.

Collagen provides the major structural strength for the fetal membranes. Loss of collagen and structural strength leading to rupture of the membranes is mediated by matrix metalloproteinases (MMPs), a large family of enzymes that act to degrade collagen and remodel tissue. Matrix metalloproteinase types 1 and 8 are collagenases that act to degrade collagen types I, II, and III. The activity of MMPs is regulated at several levels, but most importantly by tissue inhibitors of MMPs (TIMPs). A balance between activators and tissue inhibitors of metalloproteinases controls metalloprotease activity. An increased ratio of MMP 9 to TIMP 1 is associated with decrease tensile strength of fetal membranes (reviewed in [67]. MMPs 1-3, 8, 9 and 14 are upregulated in the amnion and chorion and their concentrations increased in amniotic fluid in PPROM [67]. Amniotic fluid MMP 9 concentrations are increased in the amniotic fluid of patients with PPROM, and to a lesser extent in preterm birth [68]. Increased pro-MMP 9 is found overlying the cervix in term gestation.

The activity of MMP's is primarily upregulated by decidual/fetal membrane activation during Phase 2 of spontaneous parturition at term. However, in pathologic conditions associated with preterm birth, MMP production is stimulated by infection, inflammation, or decidual hemorrhage. Bacteria or bacterial products directly secrete collagenases or stimulate MMP production [69]. Pro-inflammatory cytokines such as IL-1 and TNF-alpha also increase MMP production and decrease TIMPs in cultured membranes [70]. Thrombin, generated as a result of choriodecidual hemorrhage, also increases MMP-9 production in amniochorion cultures [49]. Finally, stretching of the membranes by uterine overdistension may result in PROM by increasing interleukin-8 and MMP activity [58].

## Genetics, environment and gene-environment interactions

There is compelling evidence that a genetic predisposition to preterm birth exists [71,72]. The evidence for a genetic or epigenetic contribution to preterm birth includes: 1. twin studies that demonstrate heritability; 2. increased recurrence risks for preterm birth among women with prior preterm birth; 3. increased risks of having preterm birth for women who were themselves born preterm; 4. increased risks of preterm birth for sisters of women who have had a preterm birth; and 5. racial disparities in preterm birth that are independent of socioeconomic factors (see [71] for a review). Twin studies have indicated a heritability of 25-40% for preterm birth [73].

Genome-wide association studies, however, have failed to identify consistent candidate genes for preterm birth. This is likely due to genetic heterogeneity. Preterm birth is a complex phenotype, with many etiologic and pathophysiological pathways that likely cannot be explained by genetic variation alone. An alternative approach has been selective candidate gene studies that have focused upon single nucleotide polymorphisms (SNPs) within selected genes. It is well established that the activity of many genes may be modified by SNPs that normally occur with varied frequencies across populations. It is estimated that 10 million SNPs may regularly occur within the human genome and constitute 90% of the variation in a population. The most common patterns of these polymorphisms are currently being assessed in the International HapMap Project [74]. More than 30 SNPs have been associated with increased or decreased risks of preterm birth or preterm premature rupture of the membranes [75,76]. Single nucleotide polymorphisms associated with preterm birth have predominately been in inflammatory and tissue remodeling pathways. Important ethnic differences in SNP frequencies may help explain racial disparities in preterm birth [77].

The role of genetic variation in stillbirth has not been explored.

Various environmental exposures have also been linked to poor pregnancy outcomes. The harmful effects of smoking during pregnancy are well established and it has been causally associated with preterm delivery and stillbirth (see article 3 on interventions).

Maternal serum and umbilical cord blood levels of pesticides such as dichlorodiphenyl trichloroethane (DDT) are associated in some, but not all, studies with preterm delivery [78]. Other organophosphate pesticide metabolites are associated with preterm birth at increasing exposure levels in the later part of pregnancy [79]. Finally, air pollution (particulate matter, carbon monoxide, lead, ozone, nitrogen dioxide, and sulfur dioxide) is associated with a variety of poor birth outcomes, including preterm birth [79]. Particulate matter is associated with small increases in preterm birth risk in studies from a number of countries such as China and the United States. Evidence is insufficient to infer causality between preterm birth and other air pollutants, but available data justifies further studies [80].

Investigation of interactions between genetics and environmental exposures are increasing, but is still limited. One recent example is the interaction between tobacco smoke exposure and preterm delivery [81,82] Smoking is an important risk factor for preterm birth, but not all women exposed to the same level of smoke deliver preterm. Gene polymorphisms in cytochrome P-450 1A1 (CYP1A1) and glutathione S-transferase theta 1 (GSTT1) have been demonstrated to increase the risk of low birth weight and preterm birth among smokers, but not among non-smokers [81,82]. These genes encode for enzymes involved in metabolism and detoxification of polycyclic aromatic hydrocarbon, an important carcinogen in tobacco smoke [82]. Women who smoke are also at increased risk of preterm delivery if they have certain factor V gene variants [83]. These women are hypothesized to be more prone to the procoagulant effects of tobacco smoke. Others have examined the interaction between air pollution and maternal genetics. Exposure to high levels of particulate matter during the third trimester in the presence of specific* GSTT1* polymorphisms has been associated with a significantly increased risk of preterm delivery [84].

Similarly, gene polymorphisms for a variety of different inflammatory cytokines (e.g., tumor necrosis factor-a, interleukins-1, -6, -10, and their receptors and antagonists) have been associated with increased risks of preterm birth [76,85] that may interact with environmental or infectious exposure. As noted above, Macones et al. noted an increased risk of preterm birth among women with bacterial vaginosis who also had a TNFα-308A allele polymorphism [32].

It is likely that many more examples of gene or environmental risk factors exist. Links between adverse pregnancy outcomes and a variety of environmental exposures have been discovered with a variable degree of strength. The data available are insufficient for many more potential exposure elements—due to limited exposure, limited human health effects, or both. There is a general lack of understanding of the dynamics of exposure during pregnancy; at what points in pregnancy is the mother/fetus most vulnerable, for example. A greater understanding is needed of the basic biological mechanisms of environmental influences on pregnancy and how they may interact with factors such as maternal genetics. Animal models may provide insight, but are limited by differences in critical periods of fetal development, genetics, and species-specific differences in suscept ibility to environmental exposures. Large-scale, well-designed and controlled human studies are necessary to establish the impact of environmental exposures on pregnancy outcomes.

## Pathways to stillbirth

Stillbirth is a major contributor to perinatal mortality, with greater than 3 million stillbirths occurring annually worldwide (see article 1 [86]). Unfortunately, despite its global impact, research into etiologic mechanisms responsible for stillbirth has been hampered by the lack of standardized definitions and reporting [6]. At least 32 classification systems of stillbirth have been described [87]. Many of these have been developed for a specific purpose, and therefore differ in classifying causes, risk factors, and co-morbid conditions. Finally, the rate of "unexplained stillbirth" differs among settings depending upon available resources and interest. The etiology of stillbirths in low- and middle-income countries are frequently more difficult to ascertain. The cause of stillbirth is attributed by verbal autopsy and classified as macerated (antepartum) or fresh (intrapartum) [88] (see article 1 [86]). The rate of stillbirth is five-fold greater in low-income countries, where resources to evaluate stillbirths—such as trained birth attendants—are scarce.

**Figure 2:**
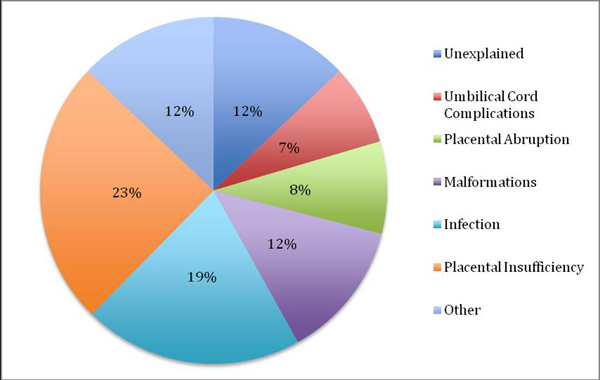
**Representative causes of stillbirth in high-income countries.** Source: Modifi ed from [90]. Reprinted from Acta Obstetricia et Gynecologica Scandinavica, 87, Varli H, Petersson K, Bottinga R, Bremme K, Hofsjo A, Holste C, Kublickas M, Norman M, Pilo C et al, The Stockholm classifi cation of stillbirth, 10, 2008, with permission from Acta Obstet Gynecol Scand.

It is likely that mechanisms responsible for preterm birth are also associated,* in extremis,* with stillbirth in many cases. In meticulous studies from Sweden, most stillbirths could be attributed to an etiology [89]. In that study, intrauterine infections, uteroplacental insufficiency, congenital malformations, placental abruption, and umbilical cord complications accounted for more than 50% of stillbirths (Figure 2). These are also important causes of preterm birth. Fewer than 20% of stillbirths could not be attributed to a specific etiology.

In contrast, up to one-third of stillbirths in low- and middle-income countries are attributed to intrapartum asphyxia, and over 50% are "unexplained." The etiology of stillbirth also varies according to gestational age [90]. One study from Canada found that between 24 and 27 weeks of gestation the most common causes of stillbirth were infection (19%), placental abruption (14%), and fetal anomalies (14%). These are also important factors for preterm birth. Beyond 28 weeks of gestation, the most frequent causes of stillbirth in this study were related to placental abruption, fetal growth restriction, and maternal illnesses [91]. However, the contribution of these pathways to stillbirth in low- and middle-income countries is largely unknown. Clearly, further research in this very important area will depend upon a uniform classification and reporting system and better ascertainment in low- and middle-income countries.

## New tools for reproductive research

One important limitation in understanding preterm births or stillbirths is the application of traditional biological methodologies to the complex process of parturition. Traditional biology attempts to explain complex phenomena by the functional properties of individual components of a complex system, a method described as "naive reductionism" [92]. Recent advances in highdimensional systems biology allow the characterization of complex processes through the global description of the components of a system and their interactions. New tools for systems biology research now exist:

• computational design (or computer assisted in-silico modeling) [93]

• genomics (characterization of genetic potential)

• transcriptomics (characterization of gene expression)

• proteomics (characterization of the protein output of gene transcription and translation)

• metabolomics (characterization of the metabolic consequences of gene expression)

Collectively, these techniques have revolutionized biologic research in the last decade. A PubMed search reveals that over 39,000 articles have been published from 1998 through 2008 utilizing these techniques (search terms "functional genomics, transcriptome, proteomics, metabolomics). These techniques, however, have been only infrequently applied to pregnancy, with approximately

**Figure 3:**
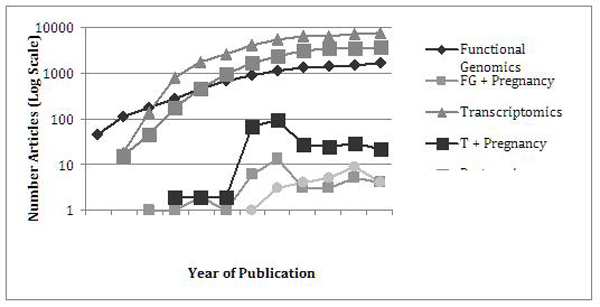
**A compilation of systems biology publications and proportion relating to pregnancy, semi-logarithmic scale.** Data source: Data abstracted from PubMed, 1997-2008. Key abstracting words “functional genomics, transcriptomics, proteomics… alone or + pregnancy”

1,000 relevant articles published in the same time period (search term "+ pregnancy"). This disparity in utilization of these powerful research tools is increasing (Figure 3) and represents a critical gap in the understanding of parturition and stillbirths.

Nonetheless, gene expression studies of the pregnant uterus have contributed to the understanding of parturition [94-99] (Table 2). The assessment of gene expression is typically measured by detection of mRNA copies produced by each gene in what are known as microarray assays. These studies differed in design, gene expression platforms, and the number of genes studied. Yet collectively, these studies demonstrate that labor is a highly complex biological process, potentially involving hundreds of genes. The number of differentially expressed genes discovered, in fact, usually depends directly upon the number sought (Table 2). The most recent study, for example, using an Affymetrix platform to ascertain activity of over 12,000 genes found 110 genes that were up-regulated, and 29 that were down-regulated in association with labor [99]. Regardless of technique, microarray analysis has led to a new understanding of labor as inherently an inflammatory process, even in the absence of infection [97,99]. Approximately 25-30% of genes—with up-regulated expression in labor—code for inflammatory proteins including chemokines, cytokines, extracellular remodeling proteins, and apoptosis. Thus, these studies have contributed to the preliminary understanding of events governing myometrial activation prior to labor.

**Table 2:** Compilation of studies comparing differential gene expression in human myometrium associated with labor

Differentially Expressed Genes Detected
			**Differentially Expressed Genes Detected**	
			
**Author & Reference**	**Platform**	**Number of Genes** **Sought on Platform**	**Up-Regulated**	**Down- Regulated**

Aguan et al., 2000 [94]	cDNA Blot	500	12	9
Chan et al., 2002 [95]	Suppressive subtraction hybridization	Not Stated	14	16
Esplin et al., 2005 [96]	cDNA microarray	6,912	22	30
Havelock et al., 2006 [97]	UniGEM-V	9,182	42	N.S.
Bukowski et al., 2006 [98]	Affymetrix	12,626	500 genes (70% Down-Regulated)
Bollapragada et al., 2009 [99]	Affymetrix	12,626	110	29

In addition to functional genomics, it is well established that the activity of many genes may be modified by SNPs that normally occur with varied frequencies across populations. More than 30 SNPs have been associated with increased or decreased risk of preterm birth or preterm premature rupture of membranes [75]. Consistent with microarray data, the majority of the SNPs associated with preterm birth are in inflammatory, apoptotic, and tissue remodeling genes [75,76] Thus, the application of systems biology, including functional genomics, transcriptomics, and SNP analysis, has contributed to a new paradigm: myometrial activation and the onset of myometrial stimulation is a highly complex genetically-controlled inflammatory process.

Proteomics has also made contributions in the understanding of abnormal parturition. Proteomics, a mass spectrometry-based technology, refers to a description of the protein complement of a system. Only 1-1.5% of the human genome codes for mRNA, and thus, leads to protein synthesis. These proteins, in turn, mediate cell-to-cell interactions in both health and disease. The major advantage of proteomics is that it most directly describes the functional output of a cell in both health and disease. Proteomics primarily aids biomarker discovery in the diagnosis of abnormal conditions of pregnancy that may contribute to prematurity. As noted by the March of Dimes, such diagnostic tools are urgently needed to facilitate timely intervention [100]. Proteomic analysis of amniotic fluid and cervical-vaginal secretions has been utilized to discover novel biomarkers for intra-amniotic infection [101,102], spontaneous preterm birth [75,103], and preterm premature rupture of the membranes [104]. Proteomic analysis of maternal urine has also identified several specific biomarkers for preeclampsia, an important cause of indicated preterm birth [75]. Additionally, comprehensive proteomic analysis has been utilized to fully characterize the protein complement of amniotic fluid and of cervical-vaginal fluid. Michaels, et al., utilizing LS/LS-MS/MS, identified 219 proteins in amniotic fluid, including 96 that were unique to amniotic fluid [105]. Similarly, Dasari, et al. identified a total of 150 unique proteins within cervical-vaginal fluid in pregnant women. Metabolism (32%) and immune response-related (22%) proteins were the major functional categories of proteins represented in the CVF proteome. A comparison of the CVF, serum, and amniotic fluid proteomes showed that 77 proteins are unique to CVF, while 56 and 17 CVF proteins also occur in serum and amniotic fluid, respectively [106]. The differential expression of proteins identified by these and other studies will likely provide the basis for diagnostic tests for many adverse pregnancy conditions including preterm birth.

Unfortunately, none of these news tools for systems biology has been applied to stillbirth. It is likely that application of these techniques in a systems biological approach will lead to significant contributions in the understanding of preterm birth and stillbirth. Because of the ability to study many processes simultaneously, however, systems biology depends upon carefully defined clinical phenotypes to avoid errors of misclassification and upon carefully prepared samples to avoid technical errors. Defining and correctly classifying differing phenotypes, or etiologies, of preterm birth or stillbirth—and appropriate collection and storage of biologic samples—are therefore critical needs and unmet gaps in systems biology and in understanding parturition.

## Identification of critical gaps in discovery sciences

**Figure 4:**
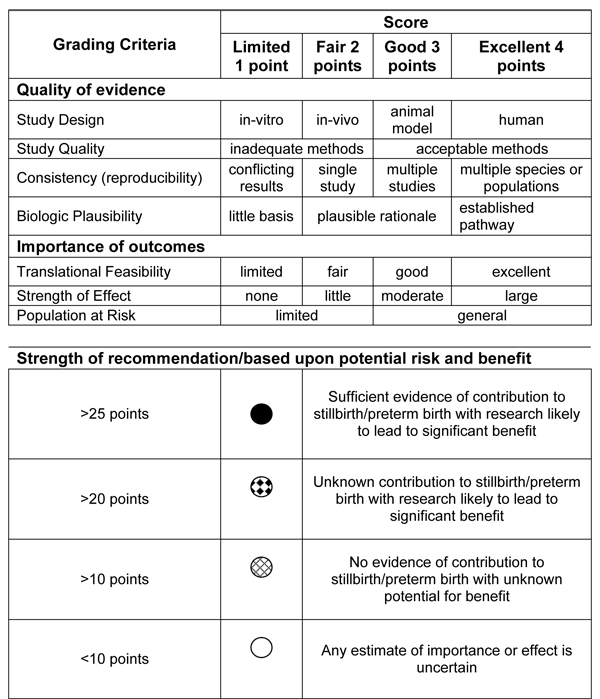
Grading criteria for evaluation of discovery science needs in preterm birth and stillbirth

In addition to these general observations of gaps and research needs, several specific gaps are noted and outlined below. In contrast to epidemiological research where elegant matrixes have been proposed to prioritize research needs and the efficacy of interventions [107,108], no such matrixes exist to assess potential benefits and risks of basic or translational science in reproductive biology. We therefore suggest an independent assessment tool to assess research gaps in basic and translational science to be applied prospectively in assessing basic and translational research needs (see Figure 4 and Additional File 1). This grading matrix, similar to CHNRI and GRADE is based upon the quality of evidence and the importance of outcomes (see Articles 1 and 3). Quality of evidence criteria include study design, soundness of methodology, consistency among or between studies, and biological rationale. A greater weight is given to human observations, consistency across species in animal models, and an established biologically plausible pathway. The importance of outcomes is based upon the ability to translate findings into clinically relevant trials, the strength of the effect, and the size of the population that may benefit. The final strength of the recommendation is therefore based upon the strength of the evidence of contribution to preterm birth or stillbirth, and the potential likelihood to yield significant benefits.

Specific gaps in basic science knowledge exist in all phases of parturition. Gaps that likely contribute to prematurity and stillbirth in which research is likely to yield significant benefit at each phase of parturition are summarized below.

### Implantation

Perturbations in implantation have been associated with habitual abortions, stillbirths, and with preeclampsia, which is an important cause of medically-indicated pre-term birth [10]. There is a critical need for a better understanding of the immunologic regulation of trophoblast invasion, and how environmental factors including chronic intrauterine infection, or exposure to environmental toxicants (e.g., cigarette smoke and insecticides) or other xenobiotic agents that may influence implantation.

### Quiescence

Progesterone is thought to be critical in maintaining uterine quiescence, and pharmacologic progesterone withdrawal is associated with increased uterine contractility. However, the influence of pollutants, xenobiotics, maternal intrauterine or systemic infections, or alterations in immune function upon progesterone (or other steroid hormone) synthesis and metabolism have not been well described, and would likely contribute to our understanding of preterm birth.

### Activation and stimulation

Activation is a complex process characterized by increasing placental production of corticotrophin releasing hormone, activation of the fetal hypothalamic-pituitary-adrenal axis, progesterone withdrawal, and increasing estrogen and prostaglandin biosynthesis. This is a complex process—microarray studies of myometrium have demonstrated differential expression of more than 100 genes in the process of activation and stimulation [99]. Many factors can stimulate or usurp the normal mechanisms of activation, including stress, infection, hemorrhage, endocrine or immunologic abnormalities, and uterine overdistension. While the role and pathophysiology of infection-induced preterm labor or preterm labor associated with hemorrhage have been well-characterized, the pathophysiology of other initiating factors has not. Specifically, there are limited or no animal models for stress or distension in preterm birth, and this represents a critical research gap. In the setting of intrauterine infection, both the fetal genotype and the maternal genotype contribute to preterm birth. However, the role of fetal versus maternal contributions to preterm birth is largely unexplored in preterm birth that is not associated with infection. Finally, the role and pathophysiology of preterm birth and especially stillbirth in the setting of systemic maternal infection is not understood. This is especially relevant given that 50 million pregnant women suffer from malaria annually, and malaria represents an important cause of stillbirth and low birth weight in low- and middle-income countries.

## Conclusion

Parturition is a continuous process beginning with implant ation and ending with involution of the uterus following birth. Active labor constitutes <0.5% of parturition. Preterm labor and stillbirth are common endpoints with multifactorial etiologies that perturb, usurp, or activate the normal processes of parturition. The physiologies of both normal and abnormal partur it ion remain poorly understood.

The following list includes critical gaps and needs in improving our understanding of these processes that are so important to survival:

• Factors regulating implantation, and the potential impact of infection or xenobiotics in normal and abnormal placentation

• Factors regulating uterine quiescence, and the role of progesterone in preventing preterm birth

• Mechanisms responsible for activation of the myometrium from a state of quiescence to a state of contractility

• Appropriate animal models to replicate pathway-specific etiologies of preterm birth or stillbirth that will contribute to rational and efficacious interventions

• The role of environmental exposure and xenobiotics in activation and stimulation of contractions

• The contributions of both the fetal and the maternal genotype in determining the clinical outcome (i.e., the phenotype) of preterm labor and stillbirth

• The role of gene:environment interactions in preterm birth and stillbirth

• The pathophysiology of common systemic infections like malaria in preterm birth and stillbirth

• The application of systems biology to better understand the complexity of parturition

In all of these critical needs, it is important to recognize that preterm birth and stillbirth are complex syndromes with many etiologies and pathways. In the past, research has been hampered by failure to recognize this heterogeneity and has led to conflicting findings. The greatest immediate needs to facilitate advances in the above gaps in knowledge include the following: carefully defined phenotypes associated with preterm birth and stillbirth; standardized definitions; uniform criteria for assessing outcomes; and the collection of biological specimens.

The next articles in this report address existing interventions [109], scale-up [110], advocacy [111], and ethics [112]. The final article presents a Global Action Agenda developed by nearly 200 global stakeholders [113]. It includes specific objectives related to normal and abnormal gestational biology, genetics, and the environment. Each is set to a specific short-, intermediate, or long-term timeline.

## Authors' contributions

The article was written by MGG. CER helped conceive of this article as part of a global report on preterm birth and stillbirth, and participated in its design, coordination, and review. TMN also helped with the coordination and review, and edited the article.

## Competing interests

The authors declare they have no competing interests.

## Supplementary Material

Additional File
