# Supplementary material for: Global report on preterm birth and stillbirth (2 of 7): discovery science
Source: BMC Pregnancy Childbirth. 2010 Feb 23;10(Suppl 1):S2. doi: 10.1186/1471-2393-10-S1-S2 (PMC2841774; doi:10.1186/1471-2393-10-S1-S2)
Supplement: Additional File [file 1471-2393-10-S1-S2-S1.doc]

**Additional File 1:** Evaluation of discovery science needs in preterm birth and stillbirth

| **Research Area** | **Context** | **Population** | **Relevance Stillbirth (1)** | **Relevance PMD (1)** | **Discovery Science** |
| --- | --- | --- | --- | --- | --- |
| **Implantation** | | | | | |
| Immune | All settings | All women |  |  | Regulation of trophopblastic invasive; Role of uterine NK cells; Role of TH1:TH2 innate immunity; Resident macrophages |
| Vascular |  |  |  |  | Spiral artery growth regulation, endothelial growth factors (e.g., VEGF) |
| Hormonal |  |  |  |  |  |
| Genetic |  |  |  |  |  |
| Infectious/Inflammation |  |  |  |  | Effect of chronic intrauterine infection upon implantation; role of viruses (e.g., LV in mice); shift in TH1:TH2 |
| Environmental |  | Both H and LMIC but potential differential effects |  |  | PCP's, etc. role on immune function; insecticides and steroid hormone analogues |
| **Uterine Quiescence** | | | | | |
| Immune | All settings | All women |  |  | Factors contributing to local/systemic immunosuppression; TH1:TH2 alterations; role of intrauterine immunoresponsive cells |
| Hormonal |  |  |  |  | Regulation of progesterone (P4) receptor isoforms; Mechanism of P4 therapy; Stable exportable P4 analogues for LMIC; Other steroid hormone influences; Regulation of the "placental clock" and corticotrophin (CRH) |
| Genetic |  |  |  |  | Microarray of genetic regulation of quiescence; Genome wide array population studies for recurrent PMD; Relative contribution of fetal vs. maternal genotype |
| Environmental |  | Both H and LMIC but potential differential effects |  |  | Xenobiotic influences on quiescence |
| **Uterine Activation (2)** | | | | | |
| Immune | All settings | All women |  |  | Role of intrauterine macrophages, NK cells; Gene regulation of effectors including MMP's, Interleukins |
| Hormonal |  |  |  |  | Activation of "placental clock"; Receptor expression in myometrium; maternal oxytocin; Role of fetal HPA axis and steroid hormone synthesis; Biomarker development for prediction; Role of contraction associated proteins (CAP's) in uterine overdistension |
| Genetic |  |  |  |  | Choriodecidual and myometrial gene expression by microarray; Protein effectors and biomarkers by proteomics; Genome wide association studies for familial or recurrent PMD; Relative contribution of fetal vs. maternal genotype |
| Infectious/Inflammatory |  |  |  |  | Pathogen recognition and signal transduction: Regulation of cytokine network; Biomarker discovery to predict pathway specific mechanisms in differing populations; Mechanisms of occult or chronic infections (e.g., periodontitis) and PMD |
| Environmental |  |  |  |  | Mechanisms of xenobiotics, pollutants, and environmental hazards |
| **Uterine Stimulation (3)** | | | | | |
| Immune | All settings | All women |  |  | Role of inflammatory mediators without infection (e.g., IL-8); pathway specific microarray |
| Hormonal |  |  |  |  | Activation of "placental clock"; Receptor expression in myometrium; maternal oxytocin; Role of fetal HPA axis; Role of CAP's in uterine overdistension |
| Genetic |  |  |  |  | Choriodecidual and myometrial gene expression by microarray; Protein effectors and biomarkers by proteomics; Genome wide association studies for familial or recurrent PMD; Relative contribution of fetal vs. maternal genotype |
| Infectious/Inflammatory |  |  |  |  | Pathogen recognition and signal transduction: Regulation of cytokine network; Biomarker discovery to predict pathway specific mechanisms in differing populations; specific patterns of pathogen colonization leading to stillbirth/PMD; role of novel non-cultivatable pathogens; Mechanisms of occult or chronic infections (e.g., periodontitis) and PMD |
| Environmental |  |  |  |  | Mechanisms of xenobiotics, pollutants, and environmental hazards |
